# Incidence of synchronous and metachronous adrenal metastases following tumor nephrectomy in renal cell cancer patients: a retrospective bi-center analysis

**DOI:** 10.1186/2193-1801-2-293

**Published:** 2013-07-01

**Authors:** Inga Peters, Milan Hora, Thomas R Herrmann, Christoph von Klot, Gerd Wegener, Petr Stransky, Ondrej Hes, Markus A Kuczyk, Axel S Merseburger

**Affiliations:** Dept. of Urology and Urologic Oncology, Hannover Medical School, Carl-Neuberg-Str. 1, 30625 Hannover, Germany; Dept. of Urology, University Hospital Plzeň, Plzeň, Czech Republic; Hannover Medical School, Tumor Center, Hannover, Germany; Dept. of Pathology, University Hospital Plzeň, Plzeň, Czech Republic

**Keywords:** Adrenal gland, Renal cell carcinoma, Synchronous metastasis, Metachronous metastasis, Laparoscopy

## Abstract

**Introduction:**

Synchronous adrenalectomy has become dispensable since retrospective studies have demonstrated no survival benefit when preoperative imaging was normal. The aim of this large bi-institutional study was to determine the appearance of synchronous and metachronous metastases to the adrenal gland as detected by computed tomography and positron emission tomography or magnetic resonance imaging with consecutive surgical removal of suspicious lesions.

**Materials and methods:**

We retrospectively reviewed the clinico-pathological records of 2720 patients from two urological centers who underwent radical or partial nephrectomy due to kidney cancer disease. Synchronous adrenalectomy was carried out in 548 of all cases (20.2%). Metachronous adrenalectomy was performed in 24 cases due to suspicious imaging in follow-up.

**Results:**

Metastatic spread in patients with synchronous adrenalectomy was found in 29/548 cases (5.3%), as suspected. In metachronous procedures positive pathological results were found in 24 of 24 cases. Among them 54% of all tumor recurrences were detected in the contralateral adrenal gland.

**Conclusions:**

In case of preoperative suspicious imaging an intraoperative frozen section should be performed. Radiological investigations are of high diagnostic value for detecting metachronous tumor growth into the adrenal gland. Surgery in this scenario should be recommended due to the high malignancy rate reported here.

## Introduction

Since the introduction of the radical nephrectomy approach for malignant kidney disease by Robson including removal of the adrenal gland more than half a century ago, this approach has been adopted towards organ- and nephron-sparing procedures (O'Malley et al. 2009).

The incidence of synchronous metastasis into the ipsilateral adrenal gland has been reported to be 2-10% (Robey and Schellhammer 1986;Winter et al. 1990). Although in advanced local disease a relevant number of patients diagnosed with renal cell cancer (RCC) are metastasized at the time of diagnosis (Hock et al. 2002), metachronous adrenal metastasis is a rare event. However, in general, metastasis to the adrenal gland is the second most common tumorous lesion in the adrenal glands after benign adrenocortical adenomas. In the case of metastatic RCC (mRCC) the incidence is from 7% to 23% in autopsy series (Wunderlich et al. 1999).

In a recent study Weight et al. demonstrated that patients do not have a benefit either in improved cancer-specific survival or in risk reduction of metachronous adrenal metastasis by performing simultaneous adrenalectomy. The authors reported synchronous ipsilateral tumor involvement of 2.2% (n=88) and a metachronous metastasis to the adrenal gland in 3.7% (n=147) after initial surgery (Weight et al. 2011). The latter finding was confirmed by other investigators, who concluded that in case of unsuspicious imaging, low tumor stage, and normal palpation during surgery the adrenal gland can be left in situ (Kobayashi et al. 2003;Kuczyk et al. 2002;Kuczyk et al. 2005). Since radiographic investigations have improved during the last decades, accounting for an increase of small adrenal lesions, it might be debatable that in case of positive radiological findings additional surgery seems to be necessary. We therefore analyzed retrospectively in a bi-center study model the number of positive pathological results (i.e. metastasis) in the adrenal gland after a tumor nephrectomy in a large cohort of patients with simultaneous adrenalectomy and with metachronous adrenalectomy due to a suspicious radiological finding in computer tomography (CT) and positron emission tomography (PET-CT) or magnetic resonance imaging (MRI). The main aim of this study is to discuss the aspects of simultaneous biopsy during radical nephrectomy surgery in case of RCC and the need of resection of a suspicious lesion in the adrenal gland in follow-up CT scans of patients with RCC history.

## Material and methods

### Study design

A retrospective bi-center study was performed. Institutional Ethical Review Boards approved the study protocol. Clinical and pathological results in the Urological Departments of the Hospital of Plzeň with 1680 patients (pts.) who underwent nephrectomy in the period 1996–2011 and 1040 pts. from Hannover Medical School (MHH) in the period 1996–2010 were included in the analysis. All patients were initially diagnosed histologically with an RCC. Laparoscopic and open radical or partial nephrectomies were performed as surgical approaches. Simultaneous adrenalectomy was performed in 232 (13.8%) patients of the Plzeň and in 316 (30.4%) patients of the MHH cohort. Along with the retrospective design of the study the lack of controlled follow-up after initial nephrectomy or partial nephrectomy of the patients was a major limitation of the study. Patients were resubmitted to surgical departments because of suspicious lesions in the adrenal glands in non-standardized routine follow-up imaging after tumor therapy and not routinely due to a randomized controlled trial.

### Patient characteristics and surgery

**Figure 1:**
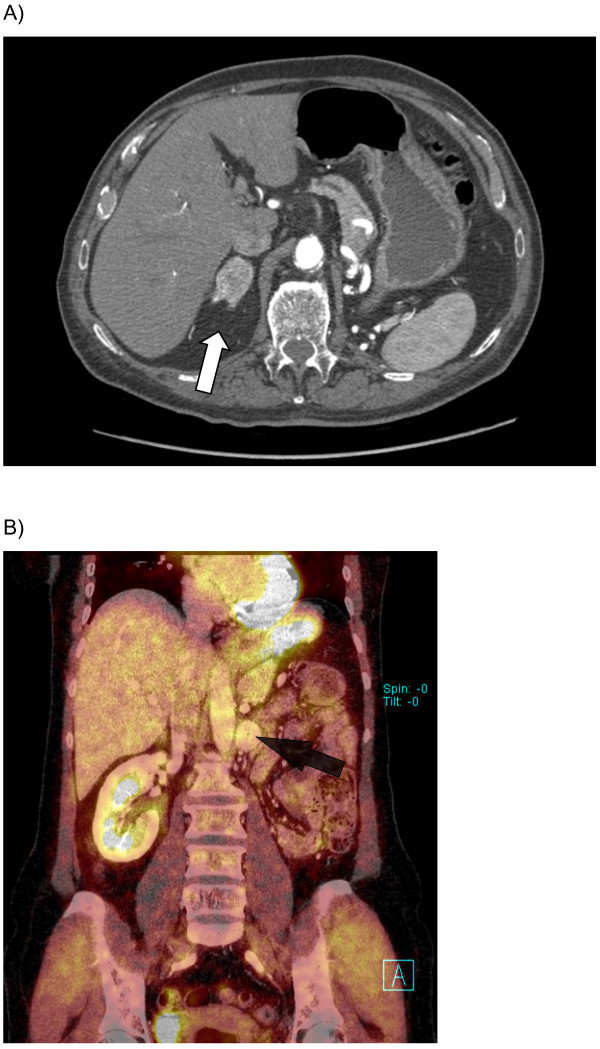
**CT (A) and PET-CT (B) scan of patients with ipsilateral metachronous metastasis after radical nephrectomy on the right (A) and left (B) side.** Arrows (white and black) mark the corresponding suspicious lesion.

**Figure 2:**
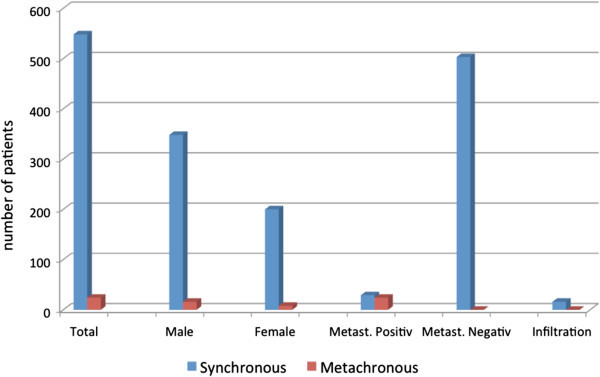
**Distribution and numbers of synchronous and metachronous metastases to adrenal gland in RCC patients during 1996-2010/2011.** Patients’ data were retrospectively analyzed regarding clinical and histopathological parameters. Blue bars indicate the synchronous and red bars the metachronous metastases.

In total 2720 patients underwent tumor surgery because of RCC. In 548 cases an adrenalectomy was simultaneously performed. The main reason for radical surgery with simultaneous adrenalectomy was a locally advanced tumor disease (T3/T4 stage) with uncertain preoperative imaging findings where differentiation of tumor involvement in contrast to periadrenal inflammation, tumor extension, or adhesion was difficult. Remaining nephrectomies were performed as adrenal-sparing surgery. Totally, in 348 men and 200 female patients the adrenal gland was resected during the procedure of nephrectomy. A metachronous adrenalectomy was performed in 24 cases, including 16 male and 8 female patients due to a suspicious finding in follow-up CT or PET-CT scan (see Figures 1 and 2). The main surgical approach for contralateral metachronous adrenalectomy was laparoscopic surgery (13/24 cases; 54%). In 2 (8.4%) cases the ipsilateral side after previous radical nephrectomy was affected and laparoscopic adrenalectomy was performed. In 13 cases the contralateral side of the primary tumor location was affected and in two cases bilateral metachronous metastatic spread was detected without any other distant metastasis. Tumors were staged according to the TNM system (Elmore et al. 2003). All resected adrenal glands were examined with regard to cancer involvement or solitary metastasis. Table 1 presents the detailed clinicopathological parameters and characteristics patients with synchronous or metachronous metastatic disease.

**Table 1:** **Clinicopathological parameters and patients’ characteristics of synchronous and metachronous adrenalectomy group (TNM 7th revision)**

Parameter	Adrenalectomy	
	**Synchronous (%) Metachronous (%)**	
**Total (n)**	548	24
**Gender (n)**		
female	200	8
male	348	16
**TNM (n, %)**		
*T1*		
A	131 (23.9)	2 (8.3)
B	88 (16.1)	7 (29.2)
*T1-T2*	3 (0.5)	3 (12.5)
*T2*		
A	47 (8.6)	1 (4.2)
B	5 (0.9)	0
*T3*		
A	135 (24.6)	5 (20.8)
B	111 (20.3)	3 (12.5)
C	6 (1.1)	0
T4	12 (2.2)	1 (4.2)
not known	10 (1.8)	2 (8.3)
N0	502 (91.6)	22 (91.7)
N1	18 (3.3)	0
N2	22 (4)	0
not known	6 (1.1)	2 (8.3)
M0	459 (83.8)	17 (70.8)
M1	82 (15)	5 (20.8)
not known	7 (1.3)	2 (8.3)
**Fuhrman grade**		
**(n, %)**		
1	122 (22.3)	4 (17.7)
2	301 (54.9)	15 (62.5)
3	97 (17.7)	4 (16.7)
4	10 (1.8)	0
not known	18 (3.3)	1 (4.2)
**Side (n, %)**		
Left	274 (50)	9 (37.5)
Right	273 (49.8)	13 (54.2)
Both	1 (0.2)	2 (8.3)

### Statistical analysis

For group comparison the chi-square test and Fisher’s exact test were used. All statistical analyses were performed using the statistical software R version 2.10.0 (Hothorn 2003). A value of *p* ≤ 0.05 was considered to be statistically significant.

## Results

**Figure 3:**
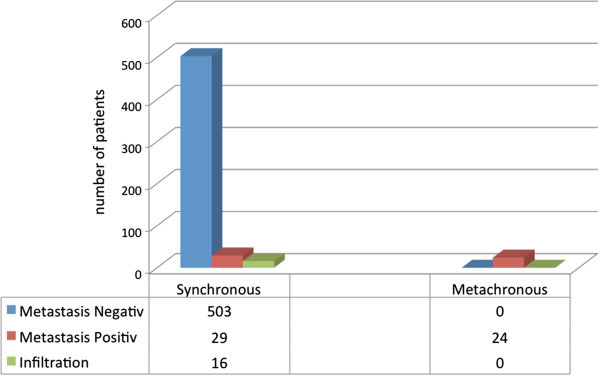
**Distribution of positive and negative pathologic results in the synchronous and metachronous adrenalectomy cohort.** Blue bars demonstrate the numbers of patients where the histopathological result was negative whereas red bars show the frequency of positive metastasis results in synchronous compared to metachronous events (*p* < 0.001, chi-square test). Green bars indicate tissues in which infiltration and no metastasis was found.

A significant statistical difference according to clinicopathological parameters of both groups (synchronous and metachronous) was not found (*p* values not shown.) Both groups were homogeneous in regard of sex, tumor status, nodal involvement or distant metastasis as well as tumor grade. However, a significant difference was found between the pathological results of resected adrenal glands. In contrast to the synchronous group, 29 out of 548 cases (5.3%) were metastasis positive, and a 100% histological positivity for clear cell renal carcinoma (ccRCC) in the metachronous group (24/24; 100%; *p* < 0.001, Figure 3) was detected. The mean time to the development of a synchronous metastasis was 52.3 months. Time to metastasis varies from a minimum of 3 months to a maximum of 157 months after initial radical surgery. A simultaneous tumor infiltration/involvement of the adrenal gland was found in 16 cases (3%) of all synchronous adrenalectomy. In 503 cases the pathological examination revealed a benign hyperplasia or cortical adenoma (Figure 3).

## Discussion

A routine ipsilateral adrenalectomy is not recommended in radical nephrectomy (Levy et al. 1998;Kuczyk, Munch et al. Munch et al. 2002) when no suspicious result is detected in imaging modalities or intraoperative findings are suspicious for tumor spread. In an earlier study we found that only a minority of RCC patients (1%) present with a solitary adrenal metastasis without other metastatic sites at the time of diagnosis (Kuczyk, Wegener et al. 2005). In cases where adrenal metastases originating from RCC were found, an advanced and disseminating disease could be found in approx. 96% (Wunderlich, Schlichter et al. 1999;Winter, Miersch et al. Miersch et al. 1990). Kobayashi et al. demonstrated in a retrospective study that there is no statistically significant difference in disease-specific survival in patients undergoing ipsilateral simultaneous adrenalectomy and patients with adrenal-sparing radical nephrectomy because of RCC (Kobayashi, Nakamura et al. 2003). We also noted a low incidence (5.3%) of synchronous adrenal metastasis. Interestingly we observed that, to our knowledge for the first time, regarding a suspicious lesion in the adrenal gland in follow-up CT scans a histological positivity (i.e. metastasis) of 100% could be found. The mean time to the development of a metachronous metastasis was 52.3 months. Both PET-CT coupled with CT or MRI are the diagnostics of choice in case of suspected adrenal lesions and in case of suspicious findings a preoperative hormonal evaluation is recommended (Sancho et al. 2012). In experienced hands a minimal invasive approach especially to patients with contralateral metachronous metastasis after previous radical nephrectomy is a feasible option (Rane et al. 2011;Abel et al. 2011).

This study supports the known results that synchronous metastasis is a rare event in RCC patients. Hence in case of suspicious lesions in follow-up imaging after adrenal-sparing radical nephrectomy a metastasis is very likely and a surgical resection should be performed. In case of advanced tumor disease at the time of diagnosis it should be discussed if in case of uncertain imaging prior to surgery (Tsui et al. 2000), an intra-operative biopsy could be helpful in deciding whether a simultaneous adrenalectomy should be performed. Otherwise postoperative hormonal dysfunction and necessity of hormonal replacement can be the result, particularly with regard to the risk of metachronous metastasis. Regarding long-term intervals to metastasis, frequent patient follow-up should be recommended. Our study suffers from bias in patient selection and retrospective data collection, which constitute study limitations. However, the presented data suggest the need of a routine and time-controlled follow-up of the patient and in case of a suspicious lesions in either CT- or MRI scan, the necessity of a surgical resection should be discussed. A prospective study with a long-term follow-up is needed to further elucidate this aspect.

## Conclusion

Synchronous adrenalectomy is not routinely recommended with nephrectomy. In case of pre-operative suspicious imaging an intraoperative frozen section and investigations by the surgeon should be performed. In case of metachronous tumor growth within the adrenal gland, PET-CT/CT or MRI should be performed. In radiological positive cases, a surgical resection of this lesion due to its high probability of malignancy should be discussed.

Furthermore, frequent follow-ups of RCC patients should be recommended due to the long mean time of the development of a metachronous metastasis of approximately 52 months.
